# Obesity stigma in Germany and the United States – Results of population surveys

**DOI:** 10.1371/journal.pone.0221214

**Published:** 2019-08-20

**Authors:** Tae Jun Kim, Anna Christin Makowski, Olaf von dem Knesebeck

**Affiliations:** Department of Medical Sociology, University Medical Center Hamburg-Eppendorf, Hamburg, Germany; University of Lleida, SPAIN

## Abstract

**Introduction:**

Over the past decades, obesity stigma has become a substantial public health issue as studies have highlighted its negative consequences for mental and physical health. However, comparative studies are scarce. In this cross-national study, we focus on the following research questions: (1) Are there differences in the magnitude of public obesity stigma between Germany and the United States (US), and (2) are there differences in the associations of sociodemographic as well as experience (i.e. former obesity experience) and contact-related (i.e. contact to a person with obesity) factors with public obesity stigma between these two countries?

**Material and methods:**

National telephone surveys in Germany and the United States were conducted (total sample = 2,802) by using vignettes for the description of a person with obesity. Fat Phobia Scale, negative reactions, and desire for social distance were assessed as components of public obesity stigma. All three stigma components were examined with multilevel linear regression analyses.

**Results:**

Overall, results show that public obesity stigma is significantly more pronounced in the US than in Germany. Relationships between obesity stigma and sociodemographic as well as experience and contact-related factors remain rather inconclusive, though, in general, stronger associations are shown in the US.

**Conclusions:**

Contrary to the normalization hypothesis, findings indicate that a comparatively high prevalence of obesity like in the US is associated with a higher level of obesity stigma.

## Introduction

The term weight bias serves to explain how one’s body weight comprises the basis for unreasonable judgements, when people with obesity are accused of being lazy, undisciplined, unintelligent, weak-willed, unsuccessful and non-compliant with weight loss efforts [1–3]. By following the stigma model from Link and Phelan [4], higher body weight is not only associated with these undesirable stereotypes, but also serves as a mark for a demarcation between ‘us’ and ‘them’. It is this very distinction between two hierarchically structured groups, which then builds the foundation for discrimination and status loss of the stigmatized.

Even if the literature on obesity has accentuated biological, economic and social factors in the formation and reinforcement of obesity [3, 5–7], societal preconceptions primarily declare obesity as the outcome of one’s personal responsibility that is avertible by adopting a right lifestyle [8,9]. In this perspective, weight stigma is justified as it expresses that obesity is undesirable and thereby helps to encourage weight loss from the stigmatized [3]. A growing number of studies, however, imply that weight stigma does not only inflict stress, social isolation and poorer (physiological and psychological) health outcomes [10–13], but also promotes unhealthy lifestyle behaviors and decreased willingness to take part in physical exercise [14–16]. Therefore, tackling weight stigma is important for societal equality and has strong implications for public health, since there is evidence that obesity stigma counteracts weight loss attempts and may even initiate subsequent weight gain through increased cortisol stress response [17,18].

Although there is a growing number of studies analyzing obesity stigma [11,17], there is a lack of comparative data that assesses this phenomenon amongst countries. In order to analyze cross-national differences in obesity stigma, this study focuses on Germany and the United States (US) as countries with rather medium to high prevalence rates of obesity (Germany = 23.6%; US = 38.2%; OECD average = 19.5%) [19]. Varying prevalence rates are the basis for two rivaling hypotheses for comparative analyses of obesity stigma: On the one hand, and according to the normalization hypothesis, a higher prevalence of obesity in a country correlates with reduced obesity stigma, as it may reinforce perceptions of overweight and obesity as a common and normalized phenomenon [20]. On the other, a higher prevalence may raise the country-specific awareness of obesity as a social and public health problem [21]. Therefore, in nations with a high prevalence of overweight and obesity, the greater perceived consequences on health may generate even stronger negative attitudes towards people with obesity.

A few studies highlight that the extent of obesity stigma depends on several social and demographic factors. According to these findings, women generally hold fewer negative attitudes towards a person with obesity compared to men [22,23]. Likewise, higher age was found to be linked to lower obesity stigma [22] based on the assumption that the importance of the thinness ideal, body shape and appearance decreases with older age [24]. Additionally, research indicates that higher education is associated with lower levels of stigmatizing attitudes [25] and there is some evidence that individuals with overweight and those who have contact with other overweight people show lower levels of obesity stigma [26].

Against this background, the present analyses will focus on the following research questions: First, are there differences in the magnitude of public obesity stigma between Germany and the US? Second, are there differences in the associations of sociodemographic and experience-related factors with public obesity stigma between these two countries?

## Material and methods

### Study and sample

National telephone surveys (computer assisted telephone interview, CATI) in Germany and the US were conducted between spring and summer 2017. Both surveys were part of a collaborative project ‘Nutrition, Health and Modern Society: Germany and the USA’ conducted by the authors in cooperation with the universities of Erfurt, Leipzig and Munich as well as USUMA, a leading market and social research institute in Germany. The study samples included adults aged 18 and older, who were living in private households. In Germany, 70% of the sample was drawn from all registered private telephone numbers at random, and additional computer-generated numbers allowed for inclusion of ex-directory households (landline numbers). The other 30% of the sample consisted of randomly generated mobile phone numbers (Random Digit Dialing, RDD). In the US, a RDD sample was generated, consisting of 60% landline numbers and 40% mobile numbers. A larger proportion of mobile numbers was drawn for the US sample as more people have a mobile phone, opposed to a landline number. Overall, 1,401 women and men voluntarily participated in each country (total sample = 2,802), reflecting a response rate of about 49% and 44% in Germany and the US, respectively. These rates were calculated based on the net sample without units that were not eligible. Eligibility was defined according to the criteria for telephone samples of the American Association of Public Opinion Research (AAPOR). Main reason for non-eligibility was that units were not residential (i.e. invalid/disconnected/non-working telephone numbers or no private household). Contacted persons were informed that participation in the study was voluntary and that withdrawal from the study was possible at any time. Moreover, they were informed that the information they provide will be kept confidential, and their name will not be shared. The Ethics Commission of the Medical Association in Hamburg reviewed and approved the study (No. PV5421).

In order to collect the respondent’s stigma attitudes, a pre-recorded vignette describing a person with obesity was played from the computer for each interview. Vignettes varied according to gender (male/female), migration status (yes/no), and occupation (lawyer/cleaner) of the person with obesity, resulting in a total of eight different vignettes (for a full description of used vignettes, please refer to S1 Appendix). Vignettes were developed in German. For the English version, we used forward-backward translation procedure to assure equivalence. In terms of migration status, vignettes were adapted, because countries of origin of migrants differ between Germany and the US. In Germany, the respective person came from Turkey and has a Turkish name while in the US the country of origin was Mexico. As these were randomly assigned to the respondents, each vignette was presented to about 175 respondents in each country. For the present analyses, all vignettes were included and pooled.

### Measures

At the beginning of the interview, one of the described vignettes was presented to the respondents. Subsequent questions about stigma referred to the vignette. Three components of the stigma process were measured [4]: (1) characteristics ascribed to the presented person with obesity (stereotypes), (2) negative reactions to a person like this, and (3) the desire for social distance.

Stereotypes were assessed using the 14-items short form of the Fat Phobia Scale [27]. This instrument covers 14 pairs of adjectives on a sematic differential (e.g. industrious—lazy, strong—weak, secure–insecure), and respondents were asked to rate the person described in the vignette on a scale ranging from 1 to 5. The scale was translated into German and validated by Stein and colleagues [28]. We calculated a sum scale (ranging from 1 to 5) where higher values indicate stronger negative stereotyping and higher fat phobia. Cronbach’s Alpha of the Fat Phobia Scale was 0.76 in Germany and 0.75 in the US.

Negative emotional and cognitive reactions towards a person with obesity in the vignette were collected by six items (angry, annoyed, incomprehension, repulsive, disgust, unaesthetic). Respondents were asked to agree or disagree to the respective reaction on a four-point Likert scale. The first three items were taken from an instrument previously developed by Angermeyer et al. [29] and available in English and German. We added three items that were expected to be specific for people with obesity (“I think that’s repulsive”; “I feel disgust”; I think that’s unaesthetic”). As the six items were not previously tested, a factor analysis (principal component analyses with varimax rotation) was performed. In both countries, all items loaded on one factor explaining 49.8% of the variance in the US and 51.5% in Germany. To test for the variance of the items on emotional reactions, Harman’s single factor test was performed showing that there wasn’t an indication for impending common method bias. For the analysis, a sum score ranging from 6 to 24 was computed. Cronbach’s Alpha of the negative reaction scale was 0.80 in Germany and 0.79 in the US.

Desire for social distance was measured by a scale developed by Link and Phelan [4], a modified version of the Bogardus Desire for Social Distance Scale [30]. The scale is comprised of seven items that represent various social relationships to the person described in the vignette: tenant, co-worker, neighbor, child carer, in-law, and a person one would recommend for a job or person of the same social circle. Respondents were asked to specify whether they would accept the person with obesity for each social relationship on a four-point Likert scale (ranging from 1 ‘totally agree’ to 4 ‘totally disagree’). A sum score (range from 7–28) was computed to assess the respondents’ overall desire for social distance. Cronbach’s Alpha of the desire for social distance scale was 0.85 in Germany and 0.86 in the US.

Gender and age were included as socio-demographic variables. For the multivariate analyses, education (classified by the International Standard Classification of Education) was also considered [31]. In terms of experience-related factors, three variables were taken into account: (1) the respondents’ experience of having been or being overweight (yes/no), (2) whether the respondents have ever experienced a disadvantage due to their own weight (yes/no), and (3) whether they have or had personal contact to family members or friends with obesity (yes/no).

### Analyses

To compare the magnitude of obesity stigma in Germany and the US, means and standard deviations were reported for all three stigma components. Kolmogorov-Smirnov Tests and Shapiro-Wilk-Normality Tests indicated a statistical violation of the assumption of normal distribution. For this reason, non-parametric tests (Mann-Whitney) were employed to test the statistical significance of country differences.

To investigate associations between obesity stigma with socio-demographic and experience-related factors in the two countries, multi-level linear regression models were calculated. Vignettes were included as multilevel variables. In multivariate analyses, missing values were excluded listwise. For all models, unstandardized regression coefficients, 95%-confidence intervals, statistical significances, the intraclass-correlation-coefficient (ICC) and explained variances (R^2^) are provided. All comparisons of stigma items between Germany and the US as well as multilevel regression analyses were computed with R.

## Results

A description of selected characteristics of the analyzed sample is presented in Table 1. In Germany, about 10% already felt disadvantaged due to their own weight, in the US this response was elicited at twice this rate. About 73% of the German sample had contact to family members or friends with obesity (about 68% in the US).

**Table 1:** Sample characteristics.

	Germany (n = 1,390–1,401)	US (n = 1,296–1,401)
Sex (female, %)	51.1	48.8
Age (mean (standard deviation))	50.7 (18.5)	47.2 (18.5)
Educational Attainment (%)		
*Low*	68.6	1.3
*Medium*	14.5	37.4
*High*	16.4	58.0
Experienced disadvantage due to weight (yes, %)	10.1	19.9
Contact to a person with obesity (family/ friends) (yes, %)	73.4	67.8
Are you or have you ever been overweight? (yes, %)	41.8	57.9

Overall, comparisons with official national data reveal that distribution of gender and age in both samples is similar to that in the general adult population in Germany and in the US [32–34]. However, in terms of education, lower educational levels are underrepresented in the US sample, while for Germany, the distribution of educational status was comparable between sample and official statistics according to data of the United States Census Bureau [35] and the German Federal Office of Statistics [36].

When exploring country-specific differences in the Fat Phobia Scale (Table 2), significantly more pronounced negative stereotyping was reported by US participants, leading to a sum score (47.54) that was significantly higher than the sum score in Germany (45.94). Higher scores in the US were found for the descriptions of: lazy, no will power, poor self-control, inactive, weak, insecure and low self-esteem. Higher scores in Germany were found for unattractive and shapeless. Regarding the items slow, having no endurance, self-indulgent, likes food and overeats, there were no statistically significant differences between Germany and the US.

**Table 2:** Fat Phobia Scale (FPS) in Germany and the US, weighted, scale 1–5 (higher score indicates stronger negative stereotyping).

		Mean (standard deviation)		P*
		Germany (n = 1,351–1,380)	US (n = 1,303–1,358)	
Industrious	Lazy	2.65 (0.96)	2.99 (1.12)	*< 0*.*001*
Secure	Insecure	2.96 (1.14)	3.25 (1.21)	*< 0*.*001*
High self-esteem	Low self-esteem	2.98 (1.11)	3.30 (1.22)	*< 0*.*001*
Strong	Weak	3.07 (1.03)	3.20 (1.14)	*< 0*.*001*
Good self-control	Poor self-control	3.13 (1.06)	3.53 (1.18)	*< 0*.*001*
Has will power	No will power	3.18 (1.06)	3.27 (1.22)	*0*.*002*
Self-sacrificing	Self-indulgent	3.25 (0.93)	3.31 (1.18)	0.156
Active	Inactive	3.29 (1.07)	3.47 (1.23)	*< 0*.*001*
Shapeless	Shapely	3.31 (1.21)	3.15 (1.23)	*< 0*.*001*
Fast	Slow	3.35 (1.05)	3.40 (1.12)	0.306
Attractive	Unattractive	3.36 (1.00)	3.23 (1.12)	*0*.*010*
Having endurance	Having no endurance	3.38 (1.14)	3.43 (1.73)	0.427
Undereats	Overeats	3.88 (0.94)	3.80 (1.17)	0.858
Dislikes food	Likes food	4.11 (0.92)	4.05 (1.09)	0.400
FPS, sum score (range: 14–70)		45.94 (7.18)	47.54 (7.98)	< 0.001

* Mann-Whitney-U test

By examining the second stigma component (Table 3), it was revealed that in the US, negative reactions towards people with obesity were generally stronger than in Germany (sum score: Germany = 10.04; US = 12.10). These differences were statistically significant for all single items and the sum score.

**Table 3:** Negative Reactions (NR) in Germany and the US, weighted, scale 1–4 (higher score indicates a stronger negative reaction).

	Mean (standard deviation)		P*
	Germany (n = 1,372–1,389)	US (n = 1,285–1,382)	
I react angrily	1.44 (0.70)	1.81 (0.72)	*< 0*.*001*
I feel disgust	1.44 (0.70)	1.89 (0.72)	*< 0*.*001*
I feel annoyed	1.51 (0.73)	1.94 (0.70)	*< 0*.*001*
I think that’s repulsive	1.56 (0.76)	1.95 (0.71)	*< 0*.*001*
This triggers incomprehension with me	1.95 (0.90)	2.17 (0.74)	*< 0*.*001*
I think that’s unaesthetic	2.15 (0.96)	2.38 (0.73)	*< 0*.*001*
NR, Sum score (range: 6–24)	10.04 (3.41)	12.10 (3.01)	< 0.001

* Mann-Whitney-U test.

A similar pattern was shown for the desire for social distance (Table 4): respondents from the US were significantly less willing to accept a person with obesity in the described social relationships (sum score: Germany = 12.54; US = 13.90). Only for one item (“recommend this person for a job”), a higher willingness for rejection emerged in Germany, though the mean difference was considerably small.

**Table 4:** (DSD) in Germany and the US, weighted, scale 1–4 (higher score indicates a greater desire for social distance).

	Mean (standard deviation)		P*
	Germany (n = 1,334–1,391)	US (n = 1,333–1,385)	
Colleague	1.44 (0.62)	1.80 (0.58)	*< 0*.*001*
Neighbor	1.52 (0.70)	1.80 (0.58)	*< 0*.*001*
Tenant	1.85 (0.92)	2.17 (0.75)	*< 0*.*001*
Childcare	1.86 (0.90)	2.09 (0.72)	*< 0*.*001*
In-law	1.90 (0.91)	2.01 (0.64)	*< 0*.*001*
Recommend for job	1.99 (0.90)	1.98 (0.62)	*0*.*012*
Introduce friend	1.99 (0.94)	2.05 (0.65)	*< 0*.*001*
DSD, sum score (range: 7–28)	12.54 (4.31)	13.90 (3.34)	< 0.001

* Mann-Whitney-U test.

Table 5 shows the associations of sociodemographic and experience-related factors with the Fat Phobia Scale in Germany and the US. According to multiple regression analyses, male gender and higher education were significantly associated with higher Fat Phobia scores in Germany, while in the US, these negative stereotypes towards people with obesity were more pronounced among lower educated respondents and among those who never were obese. Results from the ICC indicate that the proportion of variance in the dependent variable was unaffected by the different vignettes.

**Table 5:** Multi-level linear regression analysis (Fat Phobia Scale, FPS): Unstandardized regression coefficients (b), 95%-confidence intervals (CI-95%), and statistical significances (p).

	FPS (Germany)			FPS (US)		
* *	*B*	*CI-95%*	*p*	*b*	*CI-95%*	*p*
**Fixed Parts**						
(Intercept)	3.29	3.18 – 3.40	*<* .*001*	3.64	3.50 – 3.78	*<* .*001*
Sex (female)	-0.12	-0.18 – -0.06	*<* .*001*	-0.04	-0.11 – 0.03	.286
Age	0.01	-0.00 – 0.03	.093	0.01	-0.01 – 0.03	.455
Education (ISCED, range: 1–6)	0.02	-0.00 – 0.04	.056	-0.04	-0.06 – -0.01	.*003*
Overweight experience (yes)	-0.03	-0.09 – 0.03	.269	-0.09	-0.17 – -0.02	.*012*
Disadvantage based on weight (yes)	-0.08	-0.17 – 0.02	.123	0.04	-0.05 – 0.13	.417
Contact to person with obesity (yes)	0.04	-0.03 – 0.10	.301	-0.00	-0.08 – 0.07	.923
**Random Parts**						
ICC**_vignettes_**	0.032			0.009		
Observations	1,191			1,108		
R^2^	.042			.030		

In terms of negative reactions, the multilevel analysis in Table 6 indicates that, in Germany, both age and educational attainment were associated with higher scores towards a person with obesity. For example, an additional one year in age predicted a 0.30 increase in the negative reaction scale. Furthermore, experience of own overweight and having contact to a person with obesity were associated with a decrease in negative reactions. In the US, men, older respondents and respondents with low education expressed significantly more negative reactions towards a person with obesity. Moreover, an own overweight experience, a former disadvantage experience due to one’s own weight and having contact to a person with obesity were all significantly associated with lower scores. Again, low values of the ICC imply that the variation in negative reactions cannot be regressed to the variation in the different vignettes.

**Table 6:** Multi-level linear regression analysis (Negative Reactions, NR): Unstandardized regression coefficients (b), 95%-confidence intervals (CI-95%), and statistical significances (p).

	NR (Germany)			NR (USA)		
* *	*b*	*CI-95%*	*p*	*b*	*CI-95%*	*p*
**Fixed Parts**						
(Intercept)	10.77	10.20 – 11.34	*<* .*001*	14.61	13.99 – 15.23	*<* .*001*
Gender (female)	-0.19	-0.55 – 0.16	.288	-0.94	-1.26 – -0.63	*<* .*001*
Age	0.30	0.20 – 0.40	*<* .*001*	0.11	0.03 – 0.19	.*009*
Education (ISCED, range: 1–6)	0.16	0.03 – 0.28	.*013*	-0.29	-0.40 – -0.18	*<* .*001*
Overweight experience (yes)	-0.52	-0.90 – -0.15	.*007*	-0.78	-1.12 – -0.44	*<* .*001*
Disadvantage based on weight (yes)	0.52	-0.09 – 1.13	.094	0.67	0.26 – 1.08	.*001*
Contact to person with obesity (yes)	-1.09	-1.50 – -0.68	*<* .*001*	-0.65	-0.99 – -0.31	*<* .*001*
**Random Parts**						
ICC**_vignettes_**	0.007			0.003		
Observations	1,338			1,313		
R^2^	.038			.068		

When investigating the respondent’s desire for social distance (Table 7), analyses reveal that, in Germany, both age and contact to a person with obesity were statistically significant predictors of this scale. In specific, a one-year increase in age estimated a 0.62 point increase in the desire for social distance score, and a contact to a person with obesity reduced this score by -1.47, if compared to someone reporting no contact. For the US, age was also found to be a predictor of higher desire for social distance, though the effect was smaller when compared to Germany. Additionally, education, an own overweight experience and having contact to a person with obesity were significantly linked to lower social distance scores. According to the ICC, variations in the dependent variable could not be explained by the variation in the respective vignettes.

**Table 7:** Multi-level linear regression analysis (Desire for Social Distance, DSD): Unstandardized regression coefficients (b), 95%-confidence intervals (CI-95%), and statistical significances (p).

	DSD (Germany)			DSD (USA)		
* *	*b*	*CI-95%*	*p*	*b*	*CI-95%*	*p*
**Fixed Parts**						
(Intercept)	13.80	12.88 – 14.72	*<* .*001*	16.79	16.07 – 17.51	*<* .*001*
Sex (female)	-0.13	-0.57 – 0.30	.554	-0.45	-0.80 – -0.10	.*013*
Age	0.62	0.50 – 0.74	*<* .*001*	0.23	0.14 – 0.33	*<* .*001*
Education (ISCED, range: 1–6)	0.06	-0.09 – 0.21	.424	-0.30	-0.43 – -0.18	*<* .*001*
Overweight experience (yes)	-0.37	-0.82 – 0.09	.124	-0.65	-1.02 – -0.27	*<* .*001*
Disadvantage based on weight (yes)	0.49	-0.25 – 1.23	.200	0.37	-0.09 – 0.83	.120
Contact to person with obesity (yes)	-1.46	-1.96 – -0.96	*<* .*001*	-1.35	-1.73 – -0.97	*<* .*001*
**Random Parts**						
ICC_vignettes_	0.052			0.010		
Observations	1,326			1,293		
R^2^	.124			.060		

## Discussion

This study aimed to assess differences in the magnitude of obesity stigma in Germany and the US. According to the normalization hypothesis, a higher prevalence of obesity in a country is expected to lower stigma due to processes that view obesity as a common or normal phenomenon. Contrary to this hypothesis, our results showed that public obesity stigma was stronger in the US, where the prevalence of obesity is higher when compared to Germany. This observation, in turn, leaves more room for the contesting hypothesis that a higher prevalence of obesity may imply a stronger negative discussion and representation of fatness in media and society, and thus stronger tendencies for stigmatization and discrimination [37].

Regarding the first component of the stigma process, findings from the Fat Phobia Scale suggest that negative stereotyping was more pronounced in the US than in Germany. However, when comparing the single items of the Fat Phobia Scale between Germany and the US, some differences were of interest: In the US, scores were higher for all items that accentuated the concept of individualization and self-optimization (e.g. lazy, no will power, poor self-control, inactive, weak, insecure, low self-esteem). In contrast, higher scores in Germany were only found for the descriptor shapeless, which rather refers to the dimension of aesthetics though it cannot be completely separated from modern thoughts of self-optimization. Nonetheless, these findings suggest that there is a stronger focus on self-fulfillment in the US sample.

In terms of negative emotional and cognitive reactions towards people with obesity, scores were higher in the US, regardless of the item used. This further underlines our previous observation that Fat Phobia scores seem to be more crucial in the US. This may lead to a stronger emotional affection of the population by the ongoing public problematization of obesity.

Results from single items of the desire for social distance scale reiterate the assumption that there is a stronger articulation of obesity stigma in the US than in Germany. Except for the item “I would recommend this person for a job”, all scores were higher in the US. It is, however, interesting that country differences in scores gradually diminished the further away the social relation from oneself (here: respondent) to the stigmatized (here: person with obesity in the presented vignette). This may express that people from the US have a higher desire for social distance the stronger their own privacy is affected (e.g. person with obesity as a tenant, then as a colleague, then as a neighbor etc.).

In our second research question, we aimed to discover differences in the associations of sociodemographic and experience-related factors with obesity stigma in Germany and the US. Findings on the role of gender revealed that female respondents were generally less likely to endorse obesity stigma when compared with their male counterparts. Although this relationship was found to be inconsistent across different stigma components in Germany and the US, it overall supports the observation that men have more anti-fat attitudes than women [38].

Results furthermore indicate that both negative reactions and the desire for social distance increased with age. This was found for both countries, though it should be mentioned that the strength of the association was roughly threefold higher in Germany. These results contradict previous studies which found that higher age leads to lower stigma [22,24].

When assessing the relationship between education and obesity stigma, for the German sample, statistically significant associations were only found for negative reactions, while in the US, obesity stigma was consistently lower among respondents with higher education. Yet, empirical studies on the association between education and obesity stigma remain relatively scarce, and more research is needed to fully understand how one’s educational status may affect obesity stigma [26].We assumed that the respondent’s own overweight experience correlates with lower obesity stigma. The US data supports this assumption for all stigma components, while for Germany, a statistically significant association could only be found for negative reactions. In terms of the experience of being disadvantaged due to one’s own weight, however, results are less consistent. Hereafter, a statistically significant link was only revealed for negative reactions in the US. Interestingly, the direction of the relationship was positive, which indicates that a former experience of disadvantage leads to stronger negative reactions against a person with obesity. Some explanatory potential may lie in the argument that former experiences of disadvantage boost tendencies for self-stigma, though it remains interesting that this especially affects one’s negative reactions.

People who had contact with overweight persons in their family or circle of friends were less likely to have negative reactions and a desire for social distance than people without contact. This relationship was found for both countries, though the strength of the association was slightly higher in Germany. These findings more or less confirm the “contact hypothesis”, originally developed by Allport. According to this theoretical framing, contact between non obese people and persons with obesity reduces prejudice and may promote improved intergroup relationships [39].

When interpreting these results, some limitations have to be considered. First, response rates of about 50% were achieved, leaving a potential selection bias due to the other half of the eligible sample that either refused to participate or was not available. Although comparison with official statistics revealed a similar distribution of gender and age in our samples [32–34], lower educational levels were underrepresented in the US sample and prevalence of obesity in both samples was lower than in the data of the OECD [19]. Second, all analyses were based on cross-sectional survey data which does not allow to specify any assumptions about causality between the included variables. Third, the survey of stigma components was performed with the aid of vignettes that briefly introduced a person with obesity. To preserve comparability between different vignettes, these pre-recorded vignettes were intentionally held short on information. This certainly impedes to gain a more holistic and realistic view of the described person in the vignette, which may have been necessary for an appropriate evaluation of a social relationship between the respondents and the described person. For the present analysis, the vignettes were pooled. This combination of data referring to different stimuli can be considered a limitation. Moreover, vignettes were included as multilevel variables to adjust for the variations of obesity stigma in the different vignettes. Therefore, the observed country differences cannot be attributed to variations due to the vignettes. Finally, it has to be mentioned that all data were based on self-reports.

## Conclusions

Results of this study imply that there are strong differences in the extent of obesity stigma in Germany and the US. Obesity stigma was generally higher across all stigma components in the US. Contrary to the normalization hypothesis, stigma may intensify with the increase of obesity prevalence. In view of the global increase of obesity, a further global rise of stigma is likely [40,41]. This may build the basis for a vicious cycle of stress, additional weight gain, stigmatization and discrimination [42]. In order to tackle obesity stigma appropriately, it is necessary to focus on the social determinants that promote and influence obesity as well as to consider how obesity is problematized publicly within media, medicine and politics. Future research should therefore examine the complex interplay between stigma with social factors and specific cultural discourses (e.g. body perceptions, self-optimization) to understand and offset the stigmatization of people with obesity. This is of importance since obesity stigma triggers psychological (i.e. social isolation, stress, decreased well-being) as well as physical health adversities (i.e. promotion of unhealthy lifestyles, further weight gain) [10–13, 17,18].

## Supporting information

S1 Appendix Description of vignettes. (PDF)

S1 Questionnaire German version. (PDF)

S2 Questionnaire English version. (PDF)

S1 Dataset Data used for analysis, zipped as RData file (to use with R statistics). (ZIP)
