# Supplementary material for: Obesity stigma in Germany and the United States – Results of population surveys
Source: PLoS One. 2019 Aug 20;14(8):e0221214. doi: 10.1371/journal.pone.0221214 (PMC6701774; doi:10.1371/journal.pone.0221214)
Supplement: S1 Appendix — (PDF) [file pone.0221214.s001.pdf]

S1 Appendix: Description of vignettes

Title: Obesity stigma in the United States and in Germany – results of population surveys

Authors: Tae Jun Kim, Anna Christin Makowski, Olaf von dem Knesebeck,

Contact info: Tae Jun Kim, Department of Medical Sociology, University Medical Center Hamburg-Eppendorf, Martinistr. 42, 20246 Hamburg, Germany, Email: t.kim@uke.de

Female vignettes*:

- Diana D. is a lawyer and 46 years old. With a height of 5'5 and a weight of 200 pounds, she is severely overweight.
- Diana D. is a cleaner and 46 years old. With a height of 5'5 and a weight of 200 pounds, she is severely overweight.
- Lorena D. is a Mexican-born lawyer. She is 46 years old and immigrated to the States when she was a teenager. With a height of 5'5 and a weight of 200 pounds, she is severely overweight.
- Lorena D. is a Mexican-born cleaner. She is 46 years old and immigrated to the States when she was a teenager. With a height of 5'5 and a weight of 200 pounds, she is severely overweight.

Male vignettes*:

- John D. is a lawyer and 46 years old. With a height of 5'9 and a weight of 230 pounds, he is severely overweight.
- John D. is a janitor and 46 years old. With a height of 5'9 and a weight of 230 pounds, he is severely overweight.
- Fernando D. is a Mexican-born lawyer. He is 46 years old and immigrated to the States when he was a teenager. With a height of 5'9 and a weight of 230 pounds, he is severely overweight.
- Fernando D. is a Mexican-born janitor. He is 46 years old and immigrated to the States when he was a teenager. With a height of 5'9 and a weight of 230 pounds, he is severely overweight.

* Migration vignettes were different in the German survey: Respective persons were born in Turkey, immigrated to Germany and names were Turkish (Gülsen D. and Mustafa D.).
