# Supplementary material for: Obesity stigma in Germany and the United States – Results of population surveys
Source: PLoS One. 2019 Aug 20;14(8):e0221214. doi: 10.1371/journal.pone.0221214 (PMC6701774; doi:10.1371/journal.pone.0221214)
Supplement: S1 Questionnaire — (PDF) [file pone.0221214.s002.pdf]

S1 Questionnaire: German version

Title: Obesity stigma in the United States and in Germany – results of population surveys

Authors: Tae Jun Kim, Anna Christin Makowski, Olaf von dem Knesebeck,

Contact info: Tae Jun Kim, Department of Medical Sociology, University Medical Center Hamburg-Eppendorf,

Martinistr. 42, 20246 Hamburg, Germany, Email: t.kim@uke.de

FRAGEBOGEN (VERSION 2: 4. APRIL 2017)

TEIL A: AUFKLÄRUNG UND EINWILLIGUNG

Einleitung:

Guten Morgen/Tag/Abend. Mein Name ist XX, ich bin Mitarbeiter/in des Befragungsinstituts USUMA. Wir führen im Auftrag der Universitätsklinik Hamburg eine Untersuchung zum Thema „Gesundheit, Ernährung und körperliches Wohlbefinden" durch.

Die Ergebnisse der Befragung werden uns helfen, die Unterstützung für Menschen mit gesundheitlichen Einschränkungen und Problemen zu verbessern. Wir würden uns freuen, wenn Sie daran teilnehmen.

Die Umfrage ist freiwillig, aber es ist sehr wichtig, dass möglichst alle ausgewählten Personen daran teilnehmen. Dazu möchte ich in Ihrem Haushalt gerne eine Person befragen, die mindestens 18 Jahre alt ist.

Ihre Telefonnummer wurde durch ein wissenschaftliches Zufallsverfahren ausgewählt. Die Auswertung erfolgt anonym, also nicht in Verbindung mit Ihrem Namen, Ihrer Anschrift oder Telefonnummer. Das Interview wird ca. 25 Minuten dauern.

Wenn gefragt wird, woher wir die Telefonnummer haben, antworten Sie:

Ihre Telefonnummer wurde … (USUMA, bitte konkretisieren). Wir haben hier eine Datenbank mit Nummern vorliegen, wobei die Namen nicht erfasst werden, somit ist diese Umfrage vollständig anonym.

A2	***Sind Sie bereit, an der Befragung teilzunehmen?***
	Ja
	Ja, aber nicht jetzt (z.B. keine Zeit) -> wann kann ich Sie erneut anrufen?: Tag, Uhrzeit, ggf. unter anderer Telefonnummer: ____________
	Nein (WENN NEIN, ABBRUCH)

Herzlichen Dank!

Die Befragung ist freiwillig und alle Angaben werden gemäß den Vorgaben des Bundesdatenschutzgesetzes vertraulich behandelt. Diese werden nicht an Dritte weitergegeben und nur im Zusammenhang mit den Forschungszielen verwendet.

Geschlecht (q100)

Alter (q101)

<u>Zu Beginn</u>**:**

In dieser Studie von dem Uniklinikum Hamburg geht es speziell um Ernährung, Gewicht und Gesundheit. Hierfür hätte ich gern zunächst einige Auskünfte über Sie selbst…

TEIL B: BEFRAGUNG

Block 1: Selbstauskunft

**s1**	***Sagen Sie mir bitte wie groß Sie sind?***	Freitextangabe: ______ Höhe in cm<br>99 Keine Angabe
**s2**	***Wie viel wiegen Sie aktuell?***	Freitextangabe: ______ kg<br>99 Keine Angabe
**bmi**	***BMI errechnen***	
**s2a**	*Falls Keine (genaue) Angabe bei S2:*<br>Sollten Sie es nicht genau wissen, so geben Sie bitte an, wie viel Sie ungefähr wiegen	CATI: Kategorien anhand der erfassten Körpergröße errechnen und vorschlagen<br>99 Keine Angabe
**s3**	***Wie würden Sie Ihr Gewicht selbst einschätzen? Würden Sie sagen, Sie sind…***	01 … untergewichtig,<br>02 … normalgewichtig,<br>03 … übergewichtig, -> Filter zu S6<br>04 … stark übergewichtig? -> Filter zu S6<br>99 Keine Angabe
**s4**	***Waren Sie jemals übergewichtig?***	01 Ja<br>02 Nein<br>99 Keine Angabe
**s5**	***Wie schätzen Sie das Risiko ein, selbst übergewichtig zu werden?***	01 Sehr gering<br>02 Eher gering<br>03 Eher hoch<br>04 Sehr hoch<br>09 Weiß nicht<br>99 Keine Angabe
**s6**	***Haben Sie jemals versucht, ihr Gewicht zu reduzieren?***	01 Ja<br>02 Nein<br>99 Keine Angabe
**s7**	***Haben Sie sich je aufgrund Ihres Gewichtes benachteiligt gefühlt?***	01 Ja<br>02 Nein<br>99 Keine Angabe

Block 2: Kontakte

**k1**	***Haben oder hatten Sie persönlich Kontakte zu Menschen, die übergewichtig sind?***	01 Ja<br>02 Nein<br>99 Keine Angabe
	**(Wenn ja): Waren oder sind dies Personen aus …**	Mehrfachantworten möglich
**k21**	***… Ihrer Familie?***	01 Ja<br>02 Nein<br>99 Keine Angabe
**k22**	***… Ihrer Nachbarschaft?***	01 Ja<br>02 Nein<br>99 Keine Angabe
**k23**	***… Ihrem Freundeskreis?***	01 Ja<br>02 Nein<br>99 Keine Angabe
**k24**	***… Ihrem beruflichen oder schulischen Umfeld?***	01 Ja<br>02 Nein<br>99 Keine Angabe

VIGNETTEN

Selv1

(Weiblich)

Variable	Value	Vignettentext
selv1	1	Dagmar D. ist mit einer Körpergröße von 1,68 Meter und einem Gewicht von 90 Kilogramm stark übergewichtig. Sie ist 46 Jahre alt und von Beruf Rechtsanwältin.
selv1	2	Dagmar D. ist mit einer Körpergröße von 1,68 Meter und einem Gewicht von 90 Kilogramm stark übergewichtig. Sie ist 46 Jahre alt und von Beruf Reinigungskraft.
selv1	3	Gülsen D. ist mit einer Körpergröße von 1,68 Meter und einem Gewicht von 90 Kilogramm stark übergewichtig. Sie ist 46 Jahre alt, ist als Jugendliche aus der Türkei nach Deutschland gekommen und von Beruf Rechtsanwältin.
selv1	4	Gülsen D. ist mit einer Körpergröße von 1,68 Meter und einem Gewicht von 90 Kilogramm stark übergewichtig. Sie ist 46 Jahre alt, ist als Jugendliche aus der Türkei nach Deutschland gekommen und von Beruf Reinigungskraft.
selv1	5	Dieter D. ist mit einer Körpergröße von 1,80 Meter und einem Gewicht von 105 Kilogramm stark übergewichtig. Er ist 46 Jahre alt und von Beruf Rechtsanwalt.
selv1	6	Dieter D. ist mit einer Körpergröße von 1,80 Meter und einem Gewicht von 105 Kilogramm stark übergewichtig. Er ist 46 Jahre alt und von Beruf Hausmeister.
selv1	7	Mustafa D. ist mit einer Körpergröße von 1,80 Meter und einem Gewicht von 105 Kilogramm stark übergewichtig. Er ist 46 Jahre alt, ist als Jugendlicher aus der Türkei nach Deutschland gekommen und von Beruf Rechtsanwalt.
selv1	8	Mustafa D. ist mit einer Körpergröße von 1,80 Meter und einem Gewicht von 105 Kilogramm stark übergewichtig. Er ist 46 Jahre alt, ist als Jugendlicher aus der Türkei nach Deutschland gekommen und von Beruf Hausmeister.
VigDTM	0	Kein Migrationshintergrund
VigDTM	1	Mit Migrationshintergrund

➔ *Fragetexte jeweils nach Geschlecht/Beruf/Migrationshintergrund variieren!*

Block 3: Fat Phobia Scale

Bitte denken Sie noch einmal an das Beispiel der/des [Rechtsanwältin / Reinigungskraft / Rechtsanwalts / Hausmeisters] [Dagmar D. / Gülsen D. / Dieter D. / Mustafa D.], die/der stark übergewichtig ist. Welche Eigenschaften würden Sie [Dagmar D. / Gülsen D. / Dieter D. / Mustafa D.] zuweisen? Ich lese Ihnen jetzt immer ein Eigenschaftspaar vor. Bitte stellen Sie sich eine Skala oder ein Lineal, welches Werte von 1-5 enthält, vor. Ein Beispiel: an der 1 steht „ist faul", an der 5 steht „ist fleißig". Wo auf dieser Skala würden Sie diese Person jeweils einordnen?

CATI: Permutation der Items [Attribut faul – fleißig fixieren]

**Attribut**	**Rating**	**Attribut**	**Kann ich nicht beurteilen**
Faul	1 2 3 4 5	Fleißig	
Willensschwach	1 2 3 4 5	Willensstark	
Attraktiv	1 2 3 4 5	Unattraktiv	
Beherrscht	1 2 3 4 5	Unbeherrscht	
Schnell	1 2 3 4 5	Langsam	
Hat Ausdauer	1 2 3 4 5	Hat keine Ausdauer	
Aktiv	1 2 3 4 5	Inaktiv	
Stark	1 2 3 4 5	Schwach	
Maßlos	1 2 3 4 5	Verzichtend	
Mag Essen nicht	1 2 3 4 5	Mag Essen	
Unförmig	1 2 3 4 5	Wohlgeformt	
Isst zu wenig	1 2 3 4 5	Isst zu viel	
Unsicher	1 2 3 4 5	Sicher	
Wenig selbstbewusst	1 2 3 4 5	Sehr selbstbewusst	

Block 4: Negative Reaktionen

ER

Wie würden Sie auf einen Menschen wie die/den stark übergewichtige/n [Rechtsanwältin / Rechtsanwalt / Reinigungskraft / Hausmeister] [Dagmar D. / Gülsen D. / Dieter D. / Mustafa D.] reagieren? Sagen Sie mir bitte zu jeder der folgenden möglichen Reaktionen, inwieweit diese zutreffen. Sie haben die folgenden Antwortmöglichkeiten: „Trifft gar nicht zu", „trifft eher nicht zu", „trifft eher zu", „trifft voll und ganz zu".

CATI: Permutation der Items

	**Trifft gar nicht zu**	**Trifft eher nicht zu**	**Trifft eher zu**	**Trifft voll und ganz zu**	**Weiß nicht, keine Angabe**
A) Ich fühle mich durch sie/ihn genervt.	01	02	03	04	09
B) Ich reagiere ärgerlich.	01	02	03	04	09
C) Sie/Er löst bei mir Unverständnis aus.	01	02	03	04	09
D) Ich fühle mich abgestoßen.	01	02	03	04	09
E) Ich verspüre Ekel.	01	02	03	04	09
F) Ich finde so etwas unästhetisch.	01	02	03	04	09

Block 5: Soziale Distanz

SD	*Mich würde interessieren, wie Sie persönlich zu einem Menschen mit starkem Übergewicht wie der/dem [Rechtsanwältin / Reinigungskraft / Rechtsanwalt / Hausmeister] [Dagmar D. / Gülsen D. / Dieter D. / Mustafa D.] stehen würden. Ich lese Ihnen dazu ein paar Aussagen vor. Bitte geben Sie an, was für Sie zutreffen würde. Sie haben die folgenden Antwortmöglichkeiten: "1=trifft gar nicht zu", „2=trifft eher nicht zu", „3=trifft eher zu", "4=trifft voll und ganz zu"*<br>*CATI: Permutation der Items*					
		**Trifft gar nicht zu**	**Trifft eher nicht zu**	**Trifft eher zu**	**Trifft voll und ganz zu**	**Weiß nicht, keine Angabe**
	A) Wenn ich ein Zimmer zu vermieten hätte, würde ich jemanden mit einem solchen Problem als Untermieter nehmen.	01	02	03	04	09
	B) Ich würde so jemanden als Arbeitskollegen akzeptieren.	01	02	03	04	09
	C) Mir wäre so jemand als Nachbar recht.	01	02	03	04	09
	D) Ich würde so jemandem meine Kinder für einige Stunden zur Aufsicht anvertrauen.	01	02	03	04	09
	E) Ich wäre damit einverstanden, dass so jemand in meine Familie einheiratet.	01	02	03	04	09
	F) Ich würde eine Freundin von mir mit so jemand bekannt machen.	01	02	03	04	09
	G) Wenn einer meiner Bekannten eine Arbeitsstelle zu besetzen hätte, dann würde ich ihm so jemanden empfehlen.	01	02	03	04	09

- *Wir möchten Ihnen jetzt zum Abschluss noch ein paar Fragen zu Ihrer Person stellen.*

TEIL C: SOZIO-DEMOGRAFISCHE DATEN

~~C1~~	~~**Welches Geschlecht haben Sie?**~~<br>~~☐ Männlich ☐ Weiblich~~
~~C2~~	~~**Im welchem Jahr wurden Sie geboren?**~~
**C3-1**	**In welchem Land sind Sie geboren?**<br>*-> Filter: wenn Geburtsjahr nach 1989 [C2], dann weiter mit C4*<br>☐ in Deutschland ☐ in einem anderen Land
**C3-2**	**Haben Sie vor 1989 jemals in der damaligen DDR gelebt?**<br>☐ Ja ☐ Nein
**C4**	**Welche Staatsangehörigkeit(en) haben Sie? (Mehrfachangaben möglich)**<br>☐ Deutsch und / oder<br>☐ eine andere
**C5**	**Aus welchem Land stammen Ihre Eltern?**<br>*Hinweis: Es gelten die Grenzen des heutigen Bundesgebietes.*<br>**Ihre Mutter** ☐ Aus Deutschland oder<br>☐ einem anderen Land<br>**Ihr Vater** ☐ Aus Deutschland oder<br>☐ einem anderen Land
**C6**	**Welchen Familienstand haben Sie?**<br>Ledig ☐<br>Verheiratet (mit dem Ehepartner zusammenlebend) *-> Filter zu Frage C8* ☐<br>Verheiratet (in Trennung lebend) ☐<br>Geschieden ☐<br>Verwitwet ☐
**C7**	**Haben Sie zurzeit einen festen Partner?** ☐ ja ☐ nein
**C8**	**Wie viele Personen**, Sie selbst mit eingeschlossen, wohnen ständig in Ihrem Haushalt? Gemeint sind alle Personen, die hier gemeinsam wohnen und wirtschaften. Denken Sie bitte auch an alle im Haushalt lebenden Kinder.<br>✎ ________ Personen
**C9-0**	Wie viele Jahre sind Sie insgesamt zur Schule bzw. Hochschule gegangen?<br>✎ ________________________ Jahre
**C9-1**	Welchen **höchsten Schulabschluss** bzw. **Hochschulabschluss** haben Sie?
	☐ Noch in der Schule<br>☐ Ohne Schulabschluss abgegangen -> Filter zu C10<br>☐ Sonderschule/Förderschule -> Filter zu C10<br>☐ Haupt-/Volksschulabschluss -> Filter zu C10<br>☐ Realschulabschluss/Mittlere Reife -> Filter zu C10<br>☐ Abschluss der Polytechnischen Oberschule 10. Klasse (vor 1965: 8. Klasse) -> Filter zu C10<br>☐ Fachhochschulreife -> Filter zu C10<br>☐ Erweiterte Oberschule/Allgemeine/fachgebundene Hochschulreife/Abitur -> Filter zu C10<br>☐ Fachhochschulabschluss/Hochschulabschluss -> Filter zu C10<br>☐ Einen anderen Schulabschluss und zwar: ✎ ___________________________ -> Filter zu C10

C9-2	Welchen **höchsten Schulabschluss** streben Sie an?
	☐ Haupt-/Volksschulabschluss<br>☐ Realschulabschluss/Mittlere Reife/Fachschulreife<br>☐ Fachhochschulreife/Abschluss einer Fachoberschule<br>☐ Allgemeine/fachgebundene Hochschulreife/Abitur

C10	Welche der folgenden Angaben zur **Berufstätigkeit** trifft auf Sie zu? (nur eine Nennung möglich)
	**Sie sind zurzeit...**<br>☐ nicht berufstätig (einschließlich: arbeitslos, Null-Kurzarbeit, Vorruhestand, Rentner/in, Student/in, der/die nicht gegen Geld arbeitet, Praktikant/in)<br>☐ geringfügig beschäftigt bzw. üben einen Mini-Job (450 € pro Monat) oder 1-Euro-Job aus<br>☐ Teilzeitberufstätig (mit einer wöchentlichen Arbeitszeit von 10 bis 34 Stunden) -> Filter zu C12<br>☐ Vollzeitberufstätig (mit einer wöchentlichen Arbeitszeit von 35 Stunden und mehr) -> Filter zu C12
C11	Wenn Sie **nicht vollzeit- oder teilzeiterwerbstätig sind**: Trifft eine der folgenden Angaben auf Ihre derzeitige Situation zu?
	☐ in Rente wegen teilweiser oder voller Erwerbsminderung (EU-Rente)<br>☐ Altershalber in Rente/pensioniert<br>☐ Vorzeitig in Rente/pensioniert<br>☐ Arbeitslos gemeldet<br>☐ Mutterschafts-, Erziehungsurlaub, Elternzeit<br>☐ Schüler/in<br>☐ ausschließlich Hausfrau/Hausmann<br>☐ Umschulung/Arbeitsförderungsmaßnahme<br>☐ Soziales Jahr, Bundesfreiwilligendienst<br>☐ Auszubildende/r<br>☐ Studierende(r)
C12	Welchen Beruf üben Sie aus oder haben Sie hauptsächlich ausgeübt?<br>(Falls Sie arbeitslos bzw. berentet oder Hausfrau/Hausmann sind, beziehen Sie die Fragen auf den Beruf, den Sie zuletzt hauptsächlich ausgeübt haben)<br>✎ ____________________________________
C13	Bitte beschreiben Sie mir Ihre berufliche Tätigkeit genau…<br>*Hinweis: Es geht darum, die alltägliche Tätigkeit stichpunktartig zu beschreiben.*<br>✎ ____________________________________
C14	Hat dieser Beruf, diese Tätigkeit noch einen besonderen Namen?<br>✎ ____________________________________

C15	Wie hoch etwa ist das monatliche Haushaltsnettoeinkommen, d.h. das Nettoeinkommen, das Sie (alle zusammen) nach Abzug der Steuern und Sozialabgaben haben? Gemeint sind Einkünfte zum Beispiel aus Arbeit, Rente, Sozialhilfe, Vermietung und anderer Quellen.
	☐ unter 1.000 €<br>☐ 1.000 € bis unter 1.500 €<br>☐ 1.500 € bis unter 2.000 €<br>☐ 2.000 € bis unter 2.500 €<br>☐ 2.500 € bis unter 3.000 €<br>☐ 3.000 € bis unter 3.500 €<br>☐ Über 3.500 €<br>☐ Weiß nicht<br>☐ Keine Angabe

Damit sind wir am Ende unseres Gesprächs.

Ich bedanke mich sehr herzlich für Ihre Bereitschaft an der Befragung teilzunehmen.

Ich versichere hiermit, dass ich das Interview entsprechend den USUMA-Anweisungen durchgeführt habe.

____________________	____________________	____________________
Unterschrift<br>des Interviewers	Arbeitsort<br>des Interviewers	Datum
