# Supplementary material for: Obesity stigma in Germany and the United States – Results of population surveys
Source: PLoS One. 2019 Aug 20;14(8):e0221214. doi: 10.1371/journal.pone.0221214 (PMC6701774; doi:10.1371/journal.pone.0221214)
Supplement: S2 Questionnaire — (PDF) [file pone.0221214.s003.pdf]

S2 Questionnaire: English version

Title: Obesity stigma in the United States and in Germany – results of population surveys

Authors: Tae Jun Kim, Anna Christin Makowski, Olaf von dem Knesebeck,

Contact info: Tae Jun Kim, Department of Medical Sociology, University Medical Center Hamburg-Eppendorf,

Martinistr. 42, 20246 Hamburg, Germany, Email: t.kim@uke.de

QUESTIONNAIRE (USA)

PART A: INFORMED CONSENT

Good morning/good evening. My name is XX, an assistant at the… As part of a large international research project, we are conducting a nationwide survey on "health, nutrition and well-being" for the US.

Only if necessary or if the respondent asks what the survey is about:
Let me tell you a little more about the study. If you decide to participate, you will complete a telephone interview that will take about 25 minutes to complete. During the interview, you will be asked to share some information about yourself and your opinions on the topics "health, nutrition and well-being".

Your participation in this study is completely voluntary. The information you provide will be kept confidential, and your name will not be shared.

You may skip any question you prefer not to answer. You are under no obligation to participate, and you may withdraw from the study at any time.

However, you may enjoy participating, and your participation will allow us to better understand the perspectives of people like yourself, which may lead to improvements in health care for others.

If you are asked, from where did you get the telephone number, you answer:

US context for sampling method applied.

A2	***Are you ready to participate in this survey?***
	Yes
	Yes, but not immediately (e.g. I don't have the time) -> When can I call you again?<br>Day, time, or under another number? _______________
	No (IF NO, THEN STOP INTERVIEW)

Thank you for participating in this survey!

CATI: Ascertain Gender (C1) and age (C2)

The study from the University Medical Center Hamburg has a special interest in nutrition, weight and health. Therefore, I would like to ask you a few questions about yourself…

PART B: SURVEY

1: Personal information

**S1**	***Can you tell us your current height?***	Free text: ______ Height in feet<br>99 Not specified
**S2**	***How much do you weigh?***	Free text: ______ Weight in pounds<br>99 Not specified
**S2alt**	*If not reported in S2:*<br>If you cannot recall your weight correctly, could you give us a rough estimation? Would you say your weight is between …	CATI: Compute categories on the basis of height (underweight/normal weight/over-weight/obese)<br>99 Not specified
**S3**	***How would you evaluate your own weight? Would you say you are …***	01 … underweight,<br>02 … normal weight,<br>03 … overweight,-> direct to S6<br>04 … obese? -> direct to S6
**S4**	***Have you ever been overweight?***	01 Yes<br>02 No<br>99 Not specified
**S5**	***How would you estimate your own risk of becoming overweight?***	01 Very low<br>02 Rather low<br>03 Rather high<br>04 Very high<br>09 Don't know<br>99 Not specified
**S6**	***Have you ever tried to reduce your weight?***	01 Yes<br>02 No<br>99 Not specified
**S7**	***Have you ever felt disadvantaged because of your weight?***	01 Yes<br>02 No<br>99 Not specified

2: Social contacts (experience)

**K1**	***Do you have or did you have personal contact to peo-ple that are overweight?***	01 Yes<br>02 No<br>99 Not specified
**K2**	**(If yes): Were or are these people from your …**	*Multiple answers are possible*
**K2-1**	***… Family?***	01 Yes<br>02 No<br>99 Not specified
**K2-2**	***… Neighborhood?***	01 Yes<br>02 No<br>99 Not specified
**K2-3**	***… Circle of friends?***	01 Yes<br>02 No<br>99 Not specified
**K2-4**	***… Workplace or school environment?***	01 Yes<br>02 No<br>99 Not specified

VIGNETTES

➔ *In every interview, a single vignette is presented. Vignettes vary across gender, occupation and immigration status.*

Introduction text to the Vignette: In the following, we will present you a fictitious person:

(Female)

1. **Diana D. is a lawyer and 46 years old. With a height of 5'5 and a weight of 200 pounds, she is severely overweight.**
2. **Diana D. is a cleaner and 46 years old. With a height of 5'5 and a weight of 200 pounds, she is severely overweight.**
3. **Lorena D. is a Mexican-born lawyer. She is 46 years old and immigrated to the States when she was a teenager. With a height of 5'5 and a weight of 200 pounds, she is severely overweight.**
4. **Lorena D. is a Mexican-born cleaner. She is 46 years old and immigrated to the States when she was a teenager. With a height of 5'5 and a weight of 200 pounds, she is severely overweight.**

(Male)

5. **John D. is a lawyer and 46 years old. With a height of 5'9 and a weight of 230 pounds, he is severely overweight.**
6. **John D. is a janitor and 46 years old. With a height of 5'9 and a weight of 230 pounds, he is severely overweight.**
7. **Fernando D. is a Mexican-born lawyer. He is 46 years old and immigrated to the States when he was a teenager. With a height of 5'9 and a weight of 230 pounds, he is severely overweight.**
8. **Fernando D. is a Mexican-born janitor. He is 46 years old and immigrated to the States when he was a teenager. With a height of 5'9 and a weight of 230 pounds, he is severely overweight.**

3: FAT PHOBIA SCALE (FPS)

Bacon, J.G. et al. (2001) Fat phobia scale revisited: the short form. International Journal of Obesity 25, 252-257.

**F**	***Please recall the example of the [lawyer / cleaner OR janitor] [Diana D. / Lorena D. / John D. / Fernando D.] who is severely overweight.*** ***I will present you 14 pairs of adjectives. Please imagine this pair as a scale that ranges from 1 to 5. For instance, 1 stands for lazy, and 5 for industrious.*** You can select any number in between. ***Please choose the number closest to the adjective that you feel best describes your beliefs.*** ***CATI: Permute items [exception: pair "lazy – Industrious" always as first question]***			
	**Adjective**	**Rating**	**Adjective**	**Don't know**
	**Lazy (fixed)**	**1 2 3 4 5**	**Industrious (fixed)**	
	No will power	1 2 3 4 5	Has will power	
	Attractive	1 2 3 4 5	Unattractive	
	Good self-control	1 2 3 4 5	Poor self-control	
	Fast	1 2 3 4 5	Slow	
	Having endurance	1 2 3 4 5	Having no endurance	
	Active	1 2 3 4 5	Inactive	
	Strong	1 2 3 4 5	Weak	
	Self-indulgent	1 2 3 4 5	Self-sacrificing	
	Dislikes food	1 2 3 4 5	Likes food	
	Shapeless	1 2 3 4 5	Shapely	

	Undereats	1 2 3 4 5	Overeats	
	Insecure	1 2 3 4 5	Secure	
	Low self-esteem	1 2 3 4 5	High self-esteem	

4: Negative Reactions

ER

How would you react to a person like the [lawyer / cleaner OR janitor] [Diana D. / Lorena D. / John D. / Fernando D.] who is severely overweight? You can choose the following answers: „Totally disagree“, „disagree“, „agree“, and „Totally agree“.

CATI: Permute items

	**Totally disa-gree**	**Disagree**	**Agree**	**Totally agree**	**Don't know, not spec-ified**
A) I feel annoyed	01	02	03	04	09
B) I react angrily.	01	02	03	04	09
C) This triggers incomprehension with me.	01	02	03	04	09
D) I think that's repulsive.	01	02	03	04	09
E) I feel disgust.	01	02	03	04	09
F) I think that's unaesthetic.	01	02	03	04	09

5: Social Distance

SD

In the following I would like to know how you stand to a person like the severely overweight [lawyer / cleaner OR janitor] [Diana D. / Lorena D. / John D. / Fernando D.]? I will present you a few statements. Please state if you agree with the statements that will be presented. Again, you can choose the following answers: „Totally disagree“, „disagree“, „agree“, and „Totally agree“.

CATI: Permute items

	**Totally disa-gree**	**Disagree**	**Agree**	**Totally agree**	**Don't know, not spec-ified**
A) I would rent a room in my home to someone like this person.	01	02	03	04	09
B) I would accept someone like this person as a co-worker.	01	02	03	04	09
C) I'm fine with having someone like this person as a neighbor.	01	02	03	04	09
D) I would let someone like this person take care of my children for a couple of hours.	01	02	03	04	09
E) I'm fine with having someone like this person marry a family member of mine.	01	02	03	04	09
F) I would introduce this person to a young woman I am friendly with.	01	02	03	04	09
G) If a friend was offering a job, I would recommend someone like this person	01	02	03	04	09

- *Finally, I would like to ask á few questions to your person.*

PART C: SOCIO-DEMOGRAPHICS

**C1**	**Which gender are you?**<br>☐ Male ☐ Female
**C2**	**What's your Age?**
**C2-1**	**If refusal:** If you do not wish to provide exact details, could you instead put yourself into one of the following age categories?
	☐ 18-24 years<br>☐ 25-34 years<br>☐ 35-44 years<br>☐ 45-54 years<br>☐ 55-64 years<br>☐ 56-74 years<br>☐ 75 years or older
**C3-1**	**Where were you born?**<br>☐ in the US ☐ in another country
**C4**	**What is your nationality? (multiple answers possible)**<br>☐ US citizen or<br>☐ other
**C5**	**Which country are your parents from?**<br>**Your mother** ☐ From the US or<br>☐ Another country<br>**Your father** ☐ From the US or<br>☐ Another country
**C6**	**What is your marital status?**<br>Single ☐<br>Married (living with spouse) *Direct to C8* ☐<br>Married (living separately) ☐<br>Divorced ☐<br>Widowed ☐
**C7**	**Do you have a steady partner?** ☐ yes ☐ no
**C8**	**How many persons**, you included, are permanently living in your household?<br>✎ _________ Persons
**C16R**	**What is your race?**<br>☐ American Indian or Alaska Native<br>☐ Asian<br>☐ Black or African American<br>☐ Native Hawaiian or Other Pacific Islander<br>☐ White<br>☐ don't know<br>☐ refused

**C16E**	**Are you Hispanic, Latino/a, or Spanish origin?**<br>☐ Hispanic or Latino ☐ Not Hispanic or Latino ☐ don't know ☐ refused

C9-0	How many years of full time education have you completed,including higher education?<br>🖉 ___________________________ years
**C9-1**	What is the highest level of education that you have attained?
	☐ Still in school<br>☐ Less than high school -> *Direct to C10*<br>☐ High school -> *Direct to C10*<br>☐ Junior college -> *Direct to C10*<br>☐ Bachelor degree -> *Direct to C10*<br>☐ Graduate school -> *Direct to C10*<br>☐ Another: 🖉 ___________________________ -> *Direct to C10*
**C9-2**	What level of education are you currently planning on attaining?
	☐ High school<br>☐ Junior college<br>☐ Bachelor degree<br>☐ Graduate school

**C10**	Next, we would like to know about what you do:
C10-1	**What is your main employment status?**<br>☐ … working now for pay<br>☐ … not in paid work, including: unemployed, early retirement, pensioner, Student who does not work for money, intern → **Direct to C11**<br>Are you currently in …<br>☐ … part time employment (working between 10 and 34 hours per week) → **Direct to C12**<br>☐ … full time employment (working 35 or more hours per week) → **Direct to C12**
**C11**	If you are not working for pay, what best describes your current status?
	☐ Permanently sick or disabled<br>☐ Retired<br>☐ Unemployed and looking for a job<br>☐ School student<br>☐ Other<br>☐ Doing housework, looking after the home, children or other persons<br>☐ In compulsory military service or community service<br>☐ Apprentice or trainee<br>☐ College student
**C12**	What is/was your occupation on your main job – i.e., what is/was the name or title of your main job?<br>🖉 ___________________________________
**C13**	In your main job, what kind of activities do/did you do most of the time?<br>🖉 ___________________________________

**C14**	What does/did the organization you work/worked for mainly make or do – i.e., what kind of production/function is/was performed at your workplace?<br>✐ ________________________________
**C15**	Which of these categories includes your total annual household income before taxes?<br>*(Read list and select one)*
	☐ Under $15,000
	☐ Between $15,000 - $29,999<br>☐ Between $30,000 - $49,999<br>☐ Between $50,000 - $74,999<br>☐ Between $75,000 - $99,999<br>☐ Between $100,000 - $149,999<br>☐ Between $150,000 - $199,999<br>☐ $200,000 or more<br>☐ Don't know<br>☐ Not specified

That's it.
Thank you for your participation!

I assure that this interview was conducted according to the guidelines from XXX.

______________________	______________________	______________________
Signature of interviewer	Workplace of interviewer	Date
